# Прогнозирование наличия мутации в гене <i>MEN1</i> на основании клинического фенотипа пациентов с первичным гиперпаратиреозом

**DOI:** 10.14341/probl13322

**Published:** 2023-11-10

**Authors:** Н. Г. Мокрышева, А. К. Еремкина, А. П. Милютина, Р. Х. Салимханов, Е. А. Абойшева, Е. Е. Бибик, А. М. Горбачева, А. Р. Елфимова, Е. В. Ковалева, С. В. Попов, Г. А. Мельниченко

**Affiliations:** Национальный медицинский исследовательский центр эндокринологии; Национальный медицинский исследовательский центр эндокринологии; Национальный медицинский исследовательский центр эндокринологии; Национальный медицинский исследовательский центр эндокринологии; Национальный медицинский исследовательский центр эндокринологии; Национальный медицинский исследовательский центр эндокринологии; Национальный медицинский исследовательский центр эндокринологии; Национальный медицинский исследовательский центр эндокринологии; Национальный медицинский исследовательский центр эндокринологии; Национальный медицинский исследовательский центр эндокринологии; Национальный медицинский исследовательский центр эндокринологии

**Keywords:** первичный гиперпаратиреоз, синдром множественных эндокринных неоплазий 1 типа, молекулярно-генетическое исследование, математическая модель

## Abstract

**ОБОСНОВАНИЕ:**

ОБОСНОВАНИЕ. Своевременное направление пациента на генетическое исследование с целью исключения первичного гиперпаратиреоза (ПГПТ), ассоциированного с синдромом множественных эндокринных неоплазий 1 типа (МЭН-1), — важный фактор, определяющий тактику лечения и прогноз. В условиях ограниченной доступности генетических исследований поиск клинических маркеров, указывающих на наличие мутаций в гене MEN1, остается актуальной задачей.

**ЦЕЛЬ:**

ЦЕЛЬ. Определить диагностическую ценность особенностей клинического течения ПГПТ у молодых пациентов в прогнозировании наличия мутации в гене MEN1.

**МАТЕРИАЛЫ И МЕТОДЫ:**

МАТЕРИАЛЫ И МЕТОДЫ. На базе ФГБУ «НМИЦ эндокринологии» Минздрава России проведено одноцентровое одномоментное исследование с включением 273 пациентов с ПГПТ за период 2015–2022 гг. В соответствии с результатами генетического и лабораторно-инструментального исследования выделены 3 группы пациентов: с наличием мутаций в гене MEN1 (МЭН+, n=71), с отсутствием мутаций — с изолированным спорадическим ПГПТ (МЭН-, n=158) и с ПГПТ и сопутствующими образованиями эндокринных желез — фенокопии (ФК) МЭН-1 (ФК, n=32). Отдельно выделены подгруппы пациентов моложе 40 лет. Проведен сравнительный анализ независимых групп и подгрупп, с использованием метода логистической регрессии построена математическая модель прогнозирования вероятности наличия мутации в гене MEN1.

**РЕЗУЛЬТАТЫ:**

РЕЗУЛЬТАТЫ. Пациенты групп МЭН+ и МЭН- были сопоставимы по полу, возрасту манифестации, значениям показателей кальций-фосфорного обмена, а также осложнениям ПГПТ. В группе ФК ПГПТ манифестировал позже по сравнению другими группами (p<0,001 для всех), отмечались более низкие значения общего кальция и тенденция к более низким концентрациям интактного паратгормона. В группе МЭН+ по сравнению с МЭН- и ФК статистически значимо чаще выявлялись полигландулярное поражение околощитовидных желез, рецидивы ПГПТ, отягощенный семейный анамнез. В группах ФК и МЭН-, согласно результатам гистологического исследования, преобладали аденомы (92 и 94%), в то время как в группе МЭН+ — гиперплазированные околощитовидные железы (49%). В группе ФК не было пациентов с тремя «классическими» компонентами синдрома МЭН-1, клиническое течение ПГПТ было сходно с таковым в группе МЭН-. Различия сохранялись для выделенных подгрупп пациентов моложе 40 лет, что легло в основу построения математической модели. Уравнение логистической регрессии для предсказания вероятности наличия мутации в гене MEN1 включило восемь предикторов, диагностическая чувствительность модели составила 96%, специфичность — 98%.

**ЗАКЛЮЧЕНИЕ:**

ЗАКЛЮЧЕНИЕ. На основании проведенного анализа выделены восемь предикторов наследственного характера ПГПТ в рамках синдрома МЭН-1. Разработана математическая модель для прогнозирования у пациента мутации в гене MEN1, продемонстрировавшая высокую классификационную способность на обучающей выборке. Дальнейшее совершенствование модели будет способствовать повышению качества оказания медицинской помощи пациентам с ПГПТ.

## Abstract (en)

**BACKGROUND:**

BACKGROUND: Timely referral of patients for genetic testing to rule out MEN1-associated primary PHPT is important factor in determining treatment strategy and prognosis. In the context of the limited availability of genetic testing, the search for clinical markers indicative of MEN1 gene mutations remains an extremely relevant task.

**AIM:**

AIM: To determine the diagnostic value of clinical features of primary PHPT in young patients for predicting the presence of MEN1 gene mutations.

**MATERIALS AND METHODS:**

MATERIALS AND METHODS: A single-center, prospective study was conducted at the Endocrinology Research Centre, involving 273 patients with PHPT in the period 2015–2022. Based on the results of genetic and laboratory tests, patients were divided into three groups: those with MEN1 gene mutations (MEN+ group, n=71), those without MEN1 gene mutations — isolated sporadic PHPT (MEN- group, n=158), and patients with PHPT and associated endocrine gland disorders — MEN-1 syndrome phenocopies (PHEN group, n=32). Subgroups of patients younger than 40 years of age were also identified. Comparative analysis was performed among the independent groups and subgroups, and logistic regression analysis was used to develop a mathematical model for predicting the probability of the presence of MEN1 gene mutation.

**RESULTS:**

RESULTS: Patients in the MEN+ and MEN- groups were comparable by gender and age at manifestation, as well as calcium-phosphorus metabolism parameters and PHPT complications. In the PHEN group, PHPT manifested at older age compared to the other groups (p<0.001 for all), with lower total calcium levels and a trend toward lower iPTH concentrations. The MEN+ group had a significantly higher frequency of multiglandular parathyroid (PG) involvement, PHPT recurrence, and positive family history compared to the MEN- and PHEN groups. Histologically, adenomas predominated in the PHEN and MEN- groups (92% and 94%, respectively), whereas hyperplasia of PGs were more common in the MEN+ group (49%). None of the PHEN patients had all three «classic» components of the MEN-1 syndrome, and the clinical course of PHPT was similar to that of the MEN- group. These differences were also observed in the subgroups of patients younger than 40 years, which formed the basis for the development of a mathematical model. The logistic regression equation for predicting the probability of the presence of the MEN1 gene mutation included eight predictors, with a diagnostic sensitivity of 96% and specificity of 98%.

**CONCLUSION:**

CONCLUSION: Based on the analysis performed, eight hereditary predictors of PHPT within the MEN-1 syndrome were identified. A mathematical model was developed to predict the presence of the MEN1 gene mutation in patients, which demonstrated high classification performance on the training dataset. Further refinement of the model will help improve the quality of medical care for patients with PHPT.

## ОБОСНОВАНИЕ

Первичный гиперпаратиреоз (ПГПТ) — эндокринное заболевание, характеризующееся избыточной секрецией паратиреоидного гормона (ПТГ) при высоконормальном или повышенном уровне кальция крови вследствие первичной патологии околощитовидных желез (ОЩЖ) [1]. ПГПТ наиболее часто имеет спорадический характер, однако примерно в 5% случаев обусловлен наследственным заболеванием, наиболее часто — синдромом множественных эндокринных неоплазий 1 типа (МЭН-1) [2].

МЭН-1 — заболевание с аутосомно-доминантным типом наследования, развивающееся вследствие мутации в гене менина (MEN1). «Классическими» компонентами МЭН-1 являются образования ОЩЖ, аденогипофиза и дуодено-панкреатические нейроэндокринные опухоли (НЭО) [3]. В соответствии с европейскими клиническими рекомендациями диагноз МЭН-1 устанавливается согласно следующим критериям: клиническим — при наличии 2 и более МЭН-ассоциированных образований (опухолей ОЩЖ, НЭО желудочно-кишечного тракта (ЖКТ), аденомы гипофиза); семейным — у пациентов, имеющих одно МЭН-ассоциированное образование и родственника первой линии родства с установленной мутацией в гене MEN1; генетическим — при наличии гетерозиготной мутации в гене MEN1 [4].

ПГПТ зачастую становится первым проявлением МЭН-1 и развивается до присоединения других компонентов синдрома, что определяет высокую значимость своевременной диагностики именно МЭН-1-ассоциированного ПГПТ. Для него характерны определенные клинические особенности: более молодой возраст манифестации; полигландулярный характер поражения ОЩЖ с возможным метахронным развитием образований. Имеются данные о том, что для пациентов с МЭН-1 характерно более мягкое течение заболевания, в том числе — более низкие уровни интактного паратгормона (иПТГ) и кальция крови [5–7]. В литературе описаны также отличия в развитии костных осложнений у пациентов c МЭН-1-ассоциированным ПГПТ. Так, на момент постановки диагноза при МЭН-1 синдроме показатели минеральной плотности костей (МПК), как правило, ниже по сравнению со спорадической формой заболевания. Причины этих различий пока не установлены [8]. Необходимо отметить, что в большинстве проведенных исследований, сравнивающих клинические характеристики спорадического и МЭН-1-ассоциированного ПГПТ, группы не были сопоставимы по полу и возрасту, что потенциально искажало результаты сравнения.

Несмотря на имеющиеся клинические критерии постановки диагноза синдрома МЭН-1, самым надежным вариантом диагностики в настоящее время остается генетическое исследование. И прежде всего это связано с наличием фенокопий, то есть клинического фенотипа синдрома при отсутствии мутации в гене MEN1. Наиболее часто при фенокопиях МЭН-1 обнаруживаются аденомы гипофиза (с преобладанием СТГ-продуцирующих) и опухоли ОЩЖ. Причина сочетания нескольких МЭН-1-ассоциированных образований в этом случае остается неизвестной, однако в качестве возможных причин рассматриваются мутации в других генах (например, CDKN1B), эпигенетические изменения, не исключаются ложноотрицательные результаты генетического исследования [9] Показано, что при фенокопиях возраст манифестации компонентов МЭН-1, как правило, старше, а проявление всех трех классических компонентов синдрома наблюдается крайне редко [10, 11]. В связи с этим актуальным остается вопрос об оптимальной тактике ведения таких пациентов (в частности, необходимости регулярного скрининга, рекомендуемого пациентам с генетически подтвержденным МЭН-1).

Таким образом, основная роль в постановке диагноза принадлежит врачу-клиницисту, который должен своевременно заподозрить МЭН-1 и обеспечить правильную тактику обследования, лечения и динамического наблюдения пациента. В условиях ограниченной доступности генетического исследования особую важность приобретает поиск клинических маркеров, позволяющих с высокой долей вероятности прогнозировать наличие мутаций в гене MEN1. Это позволит более персонализированно направлять на генетическое исследование, тем самым снизить финансовую нагрузку на пациента (и потенциально — на систему здравоохранения) и при этом своевременно поставить правильный диагноз.

## ЦЕЛЬ ИССЛЕДОВАНИЯ

В связи с этим целью нашего исследования стала оценка диагностической ценности клинических особенностей течения ПГПТ у молодых пациентов для прогнозирования наличия мутации в гене MEN1.

## МАТЕРИАЛЫ И МЕТОДЫ

Согласно поставленной цели, в период с 01.10.2015 г. по 01.07.2022 г. на базе ФГБУ «НМИЦ эндокринологии» Минздрава России (далее — НМИЦ эндокринологии) было проведено одноцентровое одномоментное исследование. Дизайн представлен на рисунке 1.

**Figure:**
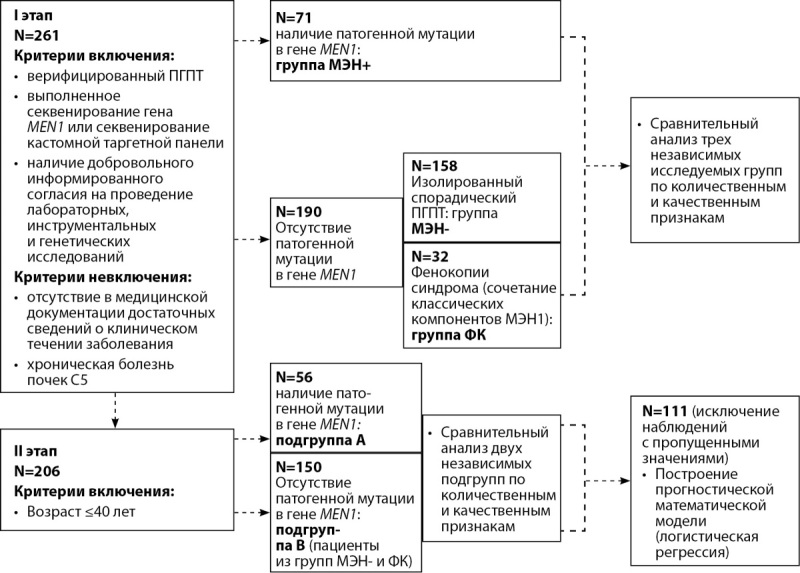
Рисунок 1. Дизайн исследования.

250 пациентам было проведено генетическое исследование в НМИЦ эндокринологии, 11 пациентам из группы МЭН+ оно выполнялось в сторонней организации. В НМИЦ эндокринологии был выполнен анализ таргетной генетической панели, включающей кодирующие регионы 378 генов, связанных с эндокринопатиями (в том числе MEN1, CASR, CDC73, RET, CDKN1B). Массивное параллельное секвенирование проводилось на платформе Illumina NextSeq 550 (Illumina, США); секвенирование по Сэнгеру осуществлялось с использованием генетического анализатора AB3500 (Thermo Fisher Scientific, США). В группе МЭН+ были получены результаты сэнгеровского секвенирования 51 пациента (секвенирование только гена MEN1), 9 пациентам был выполнен NGS-анализ кастомной панели 378 генов, связанных с эндокринопатиями. В группе МЭН- 103 пациентам было выполнено сэнгеровское секвенирование только гена MEN1, 55 пациентам — NGS-исследование панели генов. В группе ФК 15 пациентам было проведено сэнгеровское секвенирование только гена MEN1, 17 пациентам — NGS-исследование панели генов. Основаниями для назначения генетического исследования являлись подтвержденный ПГПТ у пациентов моложе 40 лет, и/или сочетание с другими компонентами синдрома МЭН-1, и/или рецидив ПГПТ, и/или наличие кровных родственников с синдромом МЭН-1 [4].

Все пациенты, включенные в представленное исследование, проходили либо амбулаторное, либо стационарное обследование и лечение в «НМИЦ эндокринологии», однако у части лабораторные и инструментальные обследования на момент манифестации ПГПТ могли быть пройдены в других медицинских учреждениях, а в карту внесены лишь их результаты. При анализе истории болезни пациентов учитывались следующие параметры: наследственный анамнез, считавшийся осложненным при наличии у родственников компонентов МЭН-1 синдрома и/или верифицированной мутации в гене MEN1; показатели минерального обмена (уровень иПТГ, кальций (Сa) общий и ионизированный, альбумин, фосфор сыворотки крови, креатинин с расчетом скорости клубочковой фильтрации (СКФ)) и суточная кальциурия на момент манифестации заболевания до проведения хирургического лечения ПГПТ, а также Ca общий, иПТГ в первые сутки после паратиреоидэктомии); результаты топической диагностики (ультразвуковое исследование (УЗИ), сцинтиграфия ОЩЖ с ОФЭКТ/КТ, компьютерная томография (КТ) ОЩЖ с контрастированием в различных комбинациях); расхождения результатов топической диагностики с интраоперационной визуализацией; наличие костных и почечных осложнений заболевания, наличие другой МЭН-1-ассоциированной эндокринной патологии. Низкоэнергетические переломы бедренных и плечевых костей, поясничных и грудных позвонков определялись по данным рентгенографии; оценка МПК проводилась по результатам рентгеновской денситометрии в поясничном отделе позвоночника, проксимальном отделе бедра и лучевой кости. Наличие осложнений со стороны почек (нефрокальциноза/нефролитиаза) устанавливалось по данным УЗИ либо КТ почек, а также по уровню СКФ (с учетом возраста и расчетной СКФ (CKD-EPI 2009)). Учитывались исходы хирургического лечения — ремиссия/рецидив/персистенция. Диагностика НЭО ЖКТ проводилась по результатам КТ или магнитно-резонансной томографии (МРТ) органов брюшной полости и забрюшинного пространства с внутривенным контрастированием, аденомы гипофиза — по результатам МРТ головного мозга, с внутривенным контрастированием при показаниях.

Статистический анализ проводился с помощью пакета прикладных программ Statistica v. 13.3 (TIBCO Software Inc., США). Сравнительный анализ трех независимых исследуемых групп по количественным признакам проведен с помощью критерия Краскела–Уоллиса с дальнейшим post-hoc анализом в случае наличия статистически значимых различий или различий на уровне статистических тенденций. Сравнительный анализ двух независимых групп по количественным признакам проведен с помощью критерия Манна–Уитни. Сравнение независимых групп по качественным признакам проводили с помощью двустороннего точного критерия Фишера. Уровень значимости (р) при проверке статистических гипотез принимался равным 0,05. Для коррекции критического уровня значимости при множественных сравнениях применялась поправка Бонферрони (р0), после чего значения р в диапазоне между рассчитанным р0 и 0,05 интерпретировались как статистическая тенденция. Для построения математической модели логистической регрессии и выполнения ROC-анализа был использован пакет прикладных программ SPSS Statistics v. 17.0 (SPSS: An IBM Company, США).

Исследования с участием людей были рассмотрены и одобрены Комитетом по этике (протокол № 1 от 17.01.2018). Письменное информированное согласие на участие в данном исследовании было предоставлено всеми участниками.

## РЕЗУЛЬТАТЫ

## Характеристика участников исследования

Общая характеристика участников исследования представлена в таблице 1.

**Table:** Таблица 1. Общая характеристика участников исследования ¹Критерий Краскела–Уоллиса.²Критерий Фишера.Поправка Бонферрони P0=0,05/23=0,002.

	МЭН+ Группа 1	МЭН- Группа 2	ФК Группа 3	р	p, post-hoc
N	Me [ Q1; Q3] /n (%)	N	Me [ Q1; Q3] /n (%)	N	Me [ Q1; Q3] /n (%)
Манифестация ПГПТ, лет	71	30 [ 25; 39]	158	33 [ 29; 37]	32	49 [ 37; 55]	<0,001¹	p1–2=0,210 p1–3<0,001 p2–3<0,001
Женщины	71	49 (69%)	158	121 (76,6%)	32	29 (90,6%)	0,052²	p1–2=0,888 p1–3=0,082 p2–3=0,331
Мужчины	71	22 (31%)	158	37 (23,4%)	32	3 (9,4%)
Аденома гипофиза в анамнезе	66	29 (43,9%)	158	0 (0%)	32	26 (79%)	<0,001²	p1–2<0,001 p1–3=0,024 p2–3<0,001
НЭО ЖКТ в анамнезе	65	31 (47,7%)	158	0 (0%)	32	3 (9,4%)	<0,001²	p1–2<0,001 p1–3=0,006 p2–3=0,026
Отягощенная наследственность	65	48 (67,6%)	158	0 (0%)	32	0 (0%)	<0,001²	p1–2<0,001 p1–3<0,001 p2–3-

Пациенты из групп МЭН+ и МЭН- были сопоставимы по возрасту манифестации заболевания (p=1,000), тогда как в группе ФК пациенты были статистически значимо старше (p<0,001 при попарном сравнении групп). Во всех трех группах преобладали женщины; различия на уровне статистической тенденции по полу пациентов были выявлены только между группами МЭН+ и ФК (табл. 1).

У большинства пациентов (79%) группы ФК были диагностированы аденомы гипофиза, в то время как НЭО ЖКТ (инсулинома, гормонально-неактивное НЭО поджелудочной железы, НЭО желудка) отмечались лишь у 3 (9,4%) больных. В группе ФК не было пациентов, имевших сочетание всех трех «классических» компонентов МЭН-1. Напротив, cреди пациентов МЭН+ с сопоставимой частотой выявлялись аденомы гипофиза и НЭО ЖКТ (43,9 и 47,7% соответственно, p=0,399), и у 12 пациентов было сочетание всех трех основных компонентов синдрома МЭН1. У 69% пациентов группы МЭН+, в отличие от пациентов других групп, отмечался осложненный наследственный анамнез.

## Параметры кальций-фосфорного обмена

Группы МЭН+ и МЭН- были сопоставимы по уровням иПТГ, Ca общего, ионизированного и скорректированного на альбумин, фосфора сыворотки крови и Ca суточной мочи. В группе ФК по сравнению с другими группами отмечались статистически более низкие значения кальциемии по уровню общего кальция, а также тенденции к более низким концентрациям ионизированного и скорректированного кальция, иПТГ. Статистически значимых различий в кальциурии, фосфатемии, уровне креатинина, а также послеоперационных концентрациях иПТГ и Са крови между тремя группами выявлено не было. Сравнительный анализ представлен в таблице 2.

**Table:** Таблица 2. Сравнительные характеристики пациентов по параметрам кальций-фосфорного обмена ¹Критерий Краскела-Уоллиса.Поправка Бонферрони P0=0,05/23=0,002.*Учитывался уровень иПТГ на первые сутки после проведения хирургического лечения.

Параметр, единицы измерения (референсный интервал)	МЭН+ Группа 1	МЭН- Группа 2	ФК Группа 3	р	p, post-hoc
N	Me [ Q1; Q3]	N	Me [ Q1; Q3]	N	Me [ Q1; Q3]
Са общий, ммоль/л (2,15–2,55)	70	2,83[ 2,71; 2,97]	158	2,79[ 2,69; 2,98]	32	2,68[ 2,62; 2,75]	<0,001¹	p1–2=0,551 p1–3<0,001 p2–3<0,001
Са ионизированный, ммоль/л (1,03–1,29)	50	1,41[ 1,32; 1,54]	114	1,36[ 1,31; 1,49]	20	1,29[ 1,26; 1,36]	0,009¹	p1–2=0,197 p1–3=0,004 p2–3=0,010
Са, скорректированный на альбумин, ммоль/л (2,15–2,55)	17	2,69[ 2,63; 2,81]	84	2,70[ 2,59; 2,88]	21	2,61[ 2,53; 2,69]	0,005¹	p1–2=0,912 p1–3=0,005 p2–3=0,002
иПТГ, пг/мл (15–65)	70	144,6[ 99,5; 220,8]	158	153,2[ 114,4; 246,0]	32	110,3[ 88,7; 156,9]	0,019¹	p1–2=0,163 p1–3=0,189 p2–3=0,006
Креатинин, мкмоль/л (50–98)	38	65,4[ 61,2; 75,6]	116	68,8[ 62,5; 76,6]	28	64,6[ 57,2; 68,5]	0,069¹	-
Фосфор, ммоль/л (0,74–1,52)	46	0,81[ 0,74; 0,97]	114	0,85[ 0,75; 0,96]	24	0,90[ 0,81; 1,08]	0,139¹	-
Кальциурия, ммоль/сут (2,5–8)	37	8,50[ 5,80; 11,72]	108	8,99[ 7,16; 12,00]	23	9,20[ 7,25; 10,06]	0,569¹	-
Послеоперационный иПТГ*, пг/мл (15–65)	41	24,52[ 11,02; 43,08]	122	20,64[ 12,66; 34,45]	16	27,25[ 19,56; 32,52]	0,270¹	-
Послеоперационный Ca общий, ммоль/л (2,15–2,55)	33	2,24[ 2,07; 2,39]	118	2,20[ 2,08; 2,32]	15	2,12[ 2,09; 2,42]	0,957¹	-

## ПГПТ-ассоциированные осложнения

Между пациентами всех трех групп не было найдено статистически значимых различий по частоте низкоэнергетических переломов, нефролитиаза либо нефрокальциноза и уровню СКФ. У пациентов с МЭН-1-ассоциированным ПГПТ по сравнению с другими группами несколько чаще отмечалось клинически значимое снижение МПК, однако различия не достигли статистической значимости (табл. 3).

**Table:** Таблица 3. Сравнительные характеристики пациентов по частоте развития осложнений ПГПТ ²Критерий Фишера.Поправка Бонферрони P0=0,05/23=0,002.

Параметр, единицы измерения (референсный интервал)	МЭН+ Группа 1	МЭН- Группа 2	ФК Группа 3	р
N	Me [ Q1; Q3] /n (%)	N	Me [ Q1; Q3] /n (%)	N	Me [ Q1; Q3] /n (%)
Нефрокальциноз/нефролитиаз	40	25 (62,5%)	129	82 (63,6%)	29	17 (58,6%)	0,864²
СКФ	38	107 [ 98; 118]	116	101 [ 91;114]	28	98,0 [ 92,8; 106,0]	0,064
Низкоэнергетические переломы	30	9 (30%)	75	18 (24%)	14	3 (21%)	0,780²
Z-score <2,0 SD или T-score <2,5 SD	35	17 (48,5%)	102	35 (33,3%)	26	9 (34,6%)	0,315²

## Основные характеристики образований околощитовидных желез

При сравнении количества образований ОЩЖ, диагностированных у пациентов по результатам предоперационной топической диагностики на момент манифестации заболевания, нами были выявлены значимые различия между группой МЭН+ и группами МЭН-, ФК (p<0,001, критерий Фишера, табл. 4). В группах МЭН- и ФК у подавляющего количества пациентов было выявлено одно образование ОЩЖ, поражение нескольких желез отмечалось редко (n=9 (5,7%) и n=1 (3,2%) соответственно). У одного пациента из группы ФК отсутствовала визуализация образования по данным УЗИ и сцинтиграфии. В группе МЭН+ полигландулярные изменения с вовлечением 2 и более ОЩЖ на дооперационном этапе выявлялись в 60,5% (n=43), при этом у 2 пациентов с мягким течением патологически измененных ОЩЖ при УЗИ не визуализировалось. Примечательно, что в группах МЭН- и ФК не было расхождений по количеству образований ОЩЖ выявленных интраоперационно и по данным предоперационной топической диагностики, в то время как в группе МЭН+ данные различия определялись для всех методов (в 50% (21/42); УЗИ vs. интраоперационная визуализация, p<0,001; сцинтиграфия vs. интраоперационная визуализация, p<0,001; МСКТ vs. интраоперационная визуализация, p<0,006).

**Table:** Таблица 4. Сравнительная характеристика групп по основным характеристикам образований ОЩЖ ²Критерий Фишера.Поправка Бонферрони P0=0,05/23=0,002.

Параметр	МЭН+ Группа 1	МЭН- Группа 2	ФК Группа 3	р	p, post-hoc
N	n (%)	N	n (%)	N	n (%)
Количество образований ≥2 по методам дооперационной топической диагностики	71	43 (60,5%)	158	9 (5,7%)	32	1 (3,2%)	<0,001²	p1–2<0,001 p1–3<0,001 p2–3=1,000
Количество образований ≥2, выявленных интраоперационно	45	40 (88,9%)	121	6 (5,0%)	17	1 (5,9%)	<0,001²	p1–2<0,001 p1–3=0,005 p2–3=1,000
Гистологическая характеристика образований	Аденома	53	25 (47,2%)	123	113 (91,9%)	16	15 (94%)	<0,001²	p1–2<0,001 p1–3 = 0,004 p2–3 = 1,000
Гиперплазия	18 (34%)	1 (0,81%)	0 (0%)
Атипическая аденома	0 (0%)	2 (1,6%)	0 (0%)
Карцинома	2 (4%)	6 (4,9%)	0 (0%)
Сочетание аденомы, гиперплазии	8 (15%)	1 (0,81%)	0 (0%)
Сочетание аденомы, атипической аденомы	0 (%)	0 (0%)	1 (6%)
Сочетание аденомы, карциномы	0 (0%)	1 (0,81%)	0 (0%)

Данные о гистологических характеристиках удаленных ОЩЖ были доступны у 192 пациентов. Группы МЭН- и ФК были сопоставимы — в обеих ожидаемо преобладали аденомы ОЩЖ, составляя 92% (113/123) и 94% (15/16) соответственно. В группе МЭН+ аденомы наблюдались в 47,2% (25/53) случаев, гиперплазия (в том числе в сочетании с аденомой) отмечена у 49,1% (26/53) больных. Карциномы ОЩЖ верифицированы у 7/123 пациентов из группы МЭН- (у 4 из них проведен расширенный генетический анализ и исключено наличие мутации в гене CDC73), у 2/53 — в группе МЭН+ (наличие мутации CDC73 исключено), злокачественного поражения ОЩЖ в группе ФК отмечено не было.

В группе МЭН+ информация об объеме первичного хирургического лечения была доступна у 59 пациентов, среди них 13 пациентам (22%) была выполнена селективная паратиреоидэктомия, 33 пациентам (55,9%) — удаление двух или трех образований, 13 (22%) пациентам — тотальная паратиреоидэктомия (удаление 4 образований).

## Характеристика послеоперационного периода

Данные по течению послеоперационного периода были доступны не у всех вошедших в исследование пациентов (табл. 5). Частота транзиторного гипопаратиреоза в послеоперационном периоде была сопоставимой в группах МЭН+ и МЭН- (36,0 и 36,5% соответственно), однако в группе ФК составила лишь 6,0%. В группе МЭН+ отсутствие ремиссии ПГПТ после проведенного хирургического лечения отмечалось чаще, чем в других группах (табл. 5).

**Table:** Таблица 5. Сравнительный анализ послеоперационного течения заболевания ²Критерий Фишера.Поправка Бонферрони P0=0,05/23=0,002.

Параметр	МЭН+ Группа 1	МЭН- Группа 2	ФК Группа 3	р	p, post-hoc
N	n (%)	N	n (%)	N	n (%)
Транзиторный послеоперационный гипопаратиреоз	50	18 (36%)	115	45 (36,5%)	17	1 (6%)	0,017²	p1–2=1,000 p1–3=0,084 p2–3=0,027
Отсутствие ремиссии ПГПТ (рецидив + персистенция)	51	19 (37%)	102	1 (1%)	16	1 (6%)	<0,001²	p1–2<0,001 p1–3=0,088 p2–3=1,000

## Характеристика пациентов молодого возраста

Для реализации второго этапа исследования из общей когорты пациентов были выделены лица молодого возраста (до 40 лет включительно). Пациенты из групп МЭН- и ФК ввиду отрицательного результата генетического анализа были объединены в одну подгруппу. Проведен сравнительный анализ подгрупп по всем основным параметрам, что и в общей когорте больных (суммарно 23 параметра) — демографические характеристики, показатели фосфорно-кальциевого обмена, осложнения ПГПТ, характеристики ОЩЖ и послеоперационного периода. Между подгруппами молодых пациентов с ПГПТ выявлены статистически значимые различия (табл. 6) по возрасту манифестации ПГПТ, наличию в анамнезе аденомы гипофиза и НЭО ЖКТ, а также отягощенной наследственности, полигландулярному поражению, гистологическим характеристикам образований ОЩЖ, отсутствию ремиссии после первичной операции. Подгруппы были сопоставимы по основным показателям фосфорно-кальциевого обмена и осложнениям ПГПТ.

**Table:** Таблица 6. Сравнительный анализ подгрупп пациентов молодого возраста ¹Критерий Манна–Уитни.²Критерий Фишера.Поправка Бонферрони P0=0,05/23=0,002.

Параметр	Подгруппа А (МЭН+)	Подгруппа В (МЭН- и ФК)	р
N	Me [ Q1; Q3] /n (%)	N	Me [ Q1; Q3] /n (%)
Манифестация ПГПТ, лет	56	28 [ 19; 32,5]	150	33 [ 28; 36]	0,001¹
Аденома гипофиза в анамнезе	52	23 (44,2%)	136	7 (5,1%)	<0,001²
НЭО ЖКТ в анамнезе	51	20 (39,2%)	129	0 (0%)	<0,001²
Отягощенная наследственность	56	34 (60,7%)	144	0 (0%)	<0,001²
Количество образований ≥2 по методам дооперационной топической диагностики	49	38 (77,6%)	150	5 (3,3%)	<0,001²
Количество образований ≥2, выявленных интраоперационно	33	28 (84,8%)	112	4 (3,6%)	<0,001²
Гистологическая характеристика образований	Аденома	40	20 (50%)	113	107 (94,7%)	<0,001²
Гиперплазия	14 (35,0%)	0 (0%)
Атипическая аденома	0 (0%)	2 (1,8%)
Карцинома	1 (2,5%)	3 (2,7%)
Сочетание аденомы, гиперплазии	5 (12,5%)	0 (0%)
Сочетание аденомы, атипической аденомы	0 (0%)	0 (0%)
Сочетание аденомы, карциномы	0 (0%)	1 (0,1%)
Отсутствие ремиссии ПГПТ (рецидив+персистенция)	40	17 (42,5%)	95	2 (2,1%)	<0,001²

## Построение математической модели для прогнозирования вероятности наличия МЭН-1-ассоциированного ПГПТ у молодых пациентов

По результатам сравнительного анализа пациентов молодого возраста были выделены основные факторы, отличающие подгруппы «ненаследственного» и МЭН-1-ассоциированного ПГПТ. С целью прогнозирования вероятности получения положительного результата генетического исследования была использована модель бинарного выбора — логистическая регрессия.

В качестве предикторов анализировались признаки, показавшие статистически значимые различия при сравнительном анализе: возраст манифестации ПГПТ (лет), наличие в анамнезе аденомы гипофиза (да — 1/нет — 0) и НЭО ЖКТ (да — 1/нет — 0), отягощенная наследственность (да — 1/нет — 0); количество образований, выявленных с помощью методов дооперационной топической диагностики (≥2–1/1–0) и количество образований, выявленных интраоперационно (≥2–1/1–0); гистологическая характеристика образований (аденома, гиперплазия, атипическая аденома, карцинома, сочетание аденомы и гиперплазии, сочетание аденомы и атипической аденомы, сочетание аденомы и карциномы), отсутствие ремиссии ПГПТ (да — 1/нет — 0). В качестве отклика (прогнозируемого признака) использовалось наличие положительного или отрицательного результата генетического исследования MEN1. После исключения наблюдений с пропущенными значениями указанных предикторов размер выборки составил 111 пациентов (22 пациента МЭН+, 89 ФК и МЭН-).

В результате было получено уравнение логистической регрессии, включающее восемь предикторов:

 (1)

где P — вероятность того, что произойдет интересующее событие, e — основание натурального логарифма 2,71,

y = -0,052x1-13,830x2+58,038x3+79,415x4-37,322x5+20,907x6-5,816x7-6,415x8+19,489

где х1–х8 — независимые признаки (x1 — возраст манифестации ПГПТ, x2 — наличие в анамнезе аденомы гипофиза, x3 — наличие в анамнезе НЭО ЖКТ, x4 — отягощенная наследственность; x5 — количество образований, выявленных с помощью методов дооперационной топической диагностики ≥2; x6 — количество образований, выявленных интраоперационно ≥2; x7 — гистологическая характеристика образований, x8 — отсутствие ремиссии ПГПТ).

Матрица классификации пациентов с генетически подтвержденным синдромом МЭН-1 с использованием полученной модели представлена в таблице 7.

**Table:** Таблица 7. Матрица классификации пациентов с генетически подтвержденным синдромом МЭН-1 с использованием полученной модели (n=111)

	Результат генетического исследования
МЭН+	МЭН - и ФК
Результат предсказания моделью	МЭН+	21	2
МЭН- и ФК	1	87

Операционные характеристики модели показали высокую классификационную способность: диагностическая чувствительность (ДЧ) 96%, 95% ДИ [ 80%; 100%]; диагностическая специфичность (ДС) 98%, 95% ДИ [ 94%; 99%]; прогностическая ценность положительного результата (ПЦПР) 91%, 95% ДИ [ 77%; 95%]; прогностическая ценность отрицательного результата (ПЦОР) 99%, 95% ДИ [ 95%; 100%].

Для графического изображения диагностической способности модели была построена ROC-кривая (рис. 2), которая отображает чувствительность и специфичность модели логистической регрессии. Площадь под кривой составила AUC = 0,983.

**Figure:**
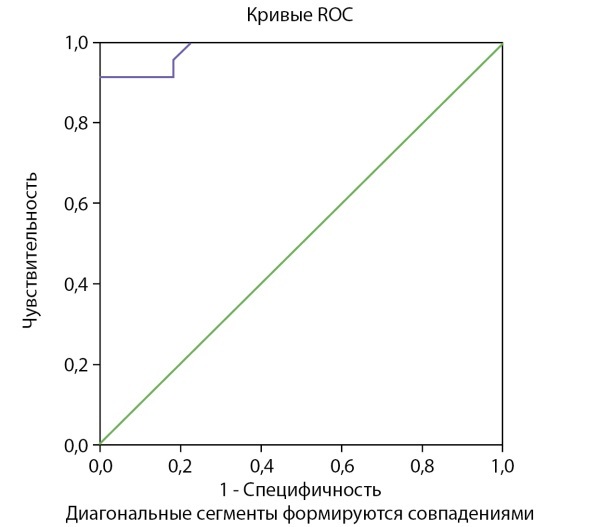
Рисунок 2. ROC-кривая полученной модели логистической регрессии.

## ОБСУЖДЕНИЕ

Для молодых пациентов с ПГПТ, ассоциированным с МЭН-1, характерно длительное бессимптомное течение. В ряде случаев умеренная гиперкальциемия может проявляться у детей и подростков, частота ее увеличивается с возрастом и уже к 50 годам повышение уровня кальция в сыворотке крови ожидается практически у всех пациентов с МЭН-1 [11]. Тем не менее при сопоставлении уровней иПТГ и кальциемии как основных показателей минерального обмена, в случае МЭН-1-ассоциированного ПГПТ наблюдались как более мягкие отклонения от референсного диапазона [12][13], так и аналогичные спорадической форме изменения [14][15]. По данным литературы, у пациентов с МЭН фиксировалось постепенное нарастание уровня иПТГ, наиболее заметное после 40 лет, в то время как для пациентов со спорадическим ПГПТ с возрастом отмечалось снижение уровней кальция крови [16][17]. В отличие от представленных работ, в нашем исследовании пациенты из групп МЭН+ и МЭН- были сопоставимы по полу и возрасту, что позволило создать оптимальные условия для сравнительного анализа именно молодой когорты больных с ПГПТ. Мы не выявили статистически значимых различий в группах МЭН+ и МЭН- по показателям фосфорно-кальциевого обмена. Однако для пациентов из группы ФК по сравнению с МЭН+ и МЭН- были характерны более низкие значения общего кальция и тенденция к более низким концентрациям иПТГ. Если принимать во внимание результаты работ Eller-Vainicher C. и соавт. и Katai M. и соавт., это могло быть обусловлено более старшим возрастом пациентов из группы ФК в сочетании с сохранной функцией почек.

Нами не было получено различий и в отношении частоты осложнений ПГПТ. Хотя у пациентов с МЭН-1-ассоциированным ПГПТ несколько чаще отмечалось снижение МПК относительно показателей, ожидаемых по возрасту, различия между группами не достигли статистической значимости (p=0,315). Наши результаты расходятся с данными других работ, где было продемонстрировано более тяжелое течение остеопороза вследствие ПГПТ у пациентов с МЭН-1 по сравнению со спорадической формой и, в ряде случаев, худшее восстановление костной ткани после хирургического лечения [18–21]. Хроническая гиперсекреция ПТГ обуславливает усиление метаболизма костной ткани, что приводит к обратимой потере массы кортикальной и трабекулярной кости из-за увеличения пространства ремоделирования и необратимой потере кортикальной кости из-за повышенной эндокортикальной резорбции [22]. Данные процессы у пациентов с МЭН-1-ассоциированным ПГПТ нередко запускаются в период набора костной массы, что и может быть причиной более ранних и тяжелых переломов в данной группе. Обсуждается вклад мутации в гене MEN1 как фактора, влияющего на созревание и функцию клеток остеогенного ряда [23]. Все три группы пациентов в нашем исследовании не различались по частоте нефролитиаза/нефрокальциноза. Lourenco D.M. и соавт. описали высокую распространенность раннего дебюта нефролитиаза у пациентов с МЭН-1 (до 86,2% у лиц моложе 30 лет), в других работах частота структурных изменений в почках была сопоставима с спорадическим ПГПТ [24][25]. Различные стадии хронической болезни почек были зарегистрированы в 19,4% случаев МЭН-1-ассоциированного ПГПТ [26].

Результаты нашей работы подтверждают, что полигландулярное поражение является одним из наиболее значимых признаков, отличающих спорадический и МЭН-1-ассоциированный ПГПТ [27]. По данным литературы, распространенность множественного поражения ОЩЖ при спорадическом ПГПТ варьирует от 7 до 33% наблюдений. Тем не менее ввиду необходимости учета ряда факторов, способных приводить к вторичному гиперпаратиреозу (ВГПТ) и гиперплазии нескольких ОЩЖ (ХБП, дефицит витамина D, мальабсорбция и др.), а также отсутствия широкого распространения скрининга генетических причин ПГПТ частота полигландулярного поражения при спорадическом ПГПТ может быть переоценена. В представленных исследованиях в морфологической структуре преобладали гиперплазии нескольких или всех ОЩЖ, реже регистрировались аденомы двух или крайне редко — трех желез [28] По нашим данным, вовлечение в патологический процесс двух и более ОЩЖ отмечалось лишь в 5,7 и 3,2% случаев в группах МЭН- и ФК соответственно и у 60,5% пациентов с МЭН-1. При этом у всех пациентов был известен генетический статус, исключались лица с ХБП С5. В группах ФК и МЭН- ожидаемо преобладали аденомы, в том числе среди лиц со множественным поражением ОЩЖ. В группе МЭН+ гиперплазии отмечались чаще, чем в двух других группах, составляя 49% случаев. Таким образом, характер поражения и гистологические характеристики образований ОЩЖ подтверждают отличия группы ФК от пациентов с МЭН-1-ассоциированным ПГПТ, и наоборот, их сходство со спорадическим заболеванием. Кроме того, в группе ФК не было пациентов со всеми тремя «классическими» компонентами синдрома МЭН-1, частота рецидивов по сравнению с МЭН+ была также меньше. Результаты нашего исследования согласуются с данными J. de Laat и др., в работе которых было проведено сравнение клинической картины у пациентов с отрицательным и положительным генетическим исследованием мутаций в гене MEN1 [29] Было установлено, что у пациентов без мутации гена менина компоненты МЭН-1 возникали в более позднем возрасте и в течение периода динамического наблюдения не отмечалось развития третьего компонента синдрома. В данном исследовании также было выявлено отсутствие снижения продолжительности жизни у пациентов, относящихся к фенокопиям МЭН-1, по сравнению с общей в популяции.

Генетическое исследование остается наиболее значимым инструментом для верификации МЭН-1 при наличии признаков, подозрительных в отношении наследственного генеза заболевания [30], однако его доступность лимитирована. Согласно проведенным исследованиям, вероятность генетической природы ПГПТ тем выше, чем меньше возраст дебюта заболевания, однако рекомендаций о генетическом скрининге в возрасте до 30, 35 или 40 лет не сформулировано. [30] Особый интерес представляет группа пациентов с ПГПТ моложе 40 лет с сочетанным полигландулярным поражением ОЩЖ и отягощенным семейным анамнезом, так как вероятность наличия синдрома при данных условиях достаточно высока [31] Учитывая данные факторы, нами была создана математическая модель, позволяющая рассчитать риск наличия мутации MEN1 именно у молодых пациентов с ПГПТ. Такая модель могла бы использоваться в клинической практике, способствуя принятию более взвешенного решения о проведении генетического анализа в ситуациях его ограниченной доступности и высокой стоимости.

Ранее модель для предсказания риска мутации в гене MEN1, разработанная на основании данных регистров МЭН-1 Нидерландов и Швеции, была предложена J. de Laat и соавт [32] В качестве факторов риска исследователи учитывали возраст пациента, ПГПТ, наличие НЭО поджелудочной железы, желудка, легких и тимуса, а также осложненный наследственный анамнез. Модель была разработана на основе анализа данных 365 пациентов с проведенным исследованием гена MEN1 и валидирована на когорте из 144 пациентов. Показатель c-statistic, отражающий предиктивную способность модели, составил 0,86 (95% ДИ 0,81–0,90) и 0,77 (95% ДИ 0,66–0,88) на тестовой и валидационной выборках соответственно, что соответствует достаточно высокой предсказательной значимости.

Предикторы в предложенной нами модели во многом схожи с моделью J. de Laat и соавт., тем не менее они были расширены за счет наличия множественного поражения ОЩЖ (≥2), выявленных до- и/или интраоперационно; гистологических характеристик и данных по рецидиву/персистенции заболевания. В нашу модель вошли дополнительные данные, которые могут быть получены у уже прооперированных пациентов, ведь нередко вопрос о возможной генетической природе заболевания возникает после операции. Как и в случае модели de Laat, нами не было получено различий по лабораторным параметрам и осложнениям ПГПТ, таким образом, они не могут быть использованы для дифференциальной диагностики наследственных и ненаследственных форм заболевания. Операционные характеристики разработанной нами модели показали достаточно высокую классификационную способность (ДЧ 96%, ДС 98%, ПЦПР 91%, ПЦОР 99%). Ввиду наличия пропусков по ряду значимых маркеров нам пришлось сократить финальную выборку пациентов до 111 человек. Однако бесспорным преимуществом стало наличие результатов генетического тестирования у всех включенных в исследование пациентов. Учитывая полученные перспективные результаты, в дальнейшем планируются расширение тестовой выборки и валидация модели.

## Ограничения исследования

Ввиду различного объема хирургического лечения возможна погрешность в результатах анализа частоты возникновения послеоперационного гипопаратиреоза и рецидива/персистенции заболевания у пациентов с МЭН-1. В ограничения нашего исследования входит недоступность данных по некоторым анализируемым параметрам в ряде случаев, у части больных — проведение инструментального и лабораторного обследования в других медицинских учреждениях.

## ЗАКЛЮЧЕНИЕ

Основными клиническими признаками, позволяющими дифференцировать МЭН-1-ассоциированный ПГПТ, являются возраст пациентов, осложненный наследственный анамнез, количество образований ОЩЖ и их гистологические характеристики, а также наличие в анамнезе рецидива ПГПТ после хирургического лечения. Данные характеристики использовались при разработке математической модели для предсказания наличия у пациента мутации в гене MEN1, продемонстрировавшей высокую классификационную способность на обучающей выборке. Дальнейшее совершенствование модели будет способствовать принятию более взвешенного решения при направлении на генетическое исследование и, таким образом, повышению качества оказания медицинской помощи пациентам с ПГПТ.

## ДОПОЛНИТЕЛЬНАЯ ИНФОРМАЦИЯ

Источники финансирования. Статья опубликована в рамках выполнения государственного задания «Оптимизация Российского электронного реестра пациентов с первичным гиперпаратиреозом» № НИОКТР 121030100032-7 при финансовой поддержке Министерства здравоохранения Российской Федерации.

Конфликт интересов. Авторы декларируют отсутствие явных и потенциальных конфликтов интересов, связанных с публикацией настоящей статьи.

Участие авторов. Мокрышева Н.Г. — существенный вклад в концепцию, дизайн исследования, внесение в рукопись существенной правки с целью повышения научной ценности статьи; Еремкина А.К. — существенный вклад в концепцию, дизайн исследования, получение, анализ данных и интерпретацию результатов, финальное редактирование текста статьи; Милютина А.П. — существенный вклад в анализ данных и интерпретацию результатов, написание статьи; Салимханов Р.Х. — существенный вклад в получение, анализ данных, написание статьи; Абойшева Е.А. — существенный вклад в получение, анализ данных, написание статьи; Бибик Е.Е. — существенный вклад в получение, анализ данных, написание статьи; Горбачева А.М. — существенный вклад в получение, анализ данных, написание статьи; Елфимова А.Р. — существенный вклад в получение, анализ данных, написание статьи; Ковалева Е.В. — существенный вклад в получение, анализ данных, написание статьи; Попов С.В. — существенный вклад в получение, анализ данных, написание статьи; Мельниченко Г.А. — существенный вклад в концепцию, дизайн исследования, внесение в рукопись существенной правки с целью повышения научной ценности статьи.

Все авторы одобрили финальную версию статьи перед публикацией, выразили согласие нести ответственность за все аспекты работы, подразумевающую надлежащее изучение и решение вопросов, связанных с точностью или добросовестностью любой части работы.
